# Remotely Estimating Aerial N Uptake in Winter Wheat Using Red-Edge Area Index From Multi-Angular Hyperspectral Data

**DOI:** 10.3389/fpls.2018.00675

**Published:** 2018-05-25

**Authors:** Bin-Bin Guo, Yun-Ji Zhu, Wei Feng, Li He, Ya-Peng Wu, Yi Zhou, Xing-Xu Ren, Ying Ma

**Affiliations:** State Key Laboratory of Wheat and Maize Crop Science, National Engineering Research Centre for Wheat, Henan Agricultural University, Zhengzhou, China

**Keywords:** winter wheat, multi-angular hyperspectral, vegetation indices, aerial N uptake, monitoring model

## Abstract

Remote sensing techniques can be efficient for non-destructive, rapid detection of wheat nitrogen (N) nutrient status. In the paper, we examined the relationships of canopy multi-angular data with aerial N uptake of winter wheat (*Triticum aestivum* L.) across different growing seasons, locations, years, wheat varieties, and N application rates. Seventeen vegetation indices (VIs) selected from the literature were measured for the stability in estimating aerial N uptake of wheat under 13 view zenith angles (VZAs) in the solar principal plane (SPP). In total, the back-scatter angles showed better VI behavior than the forward-scatter angles. The correlation coefficient of VIs with aerial N uptake increased with decreasing VZAs. The best linear relationship was integrated with the optimized common indices DIDA and DDn to examine dynamic changes in aerial N uptake; this led to coefficients of determination (*R*^2^) of 0.769 and 0.760 at the −10° viewing angle. Our novel area index, designed the modified right-side peak area index (mRPA), was developed in accordance with exploration of the spectral area calculation and red-edge feature using the equation: mRPA = (R_760_/R_600_)^1/2^ × (R_760_-R_718_). Investigating the predictive accuracy of mRPA for aerial N uptake across VZAs demonstrated that the best performance was at −10° [*R*^2^ = 0.804, *p* < 0.001, root mean square error (RMSE) = 3.615] and that the effect was relatively similar between −20° to +10° (*R*^2^ = 0.782, *p* < 0.001, RMSE = 3.805). This leads us to construct a simple model under wide-angle combinations so as to improve the field operation simplicity and applicability. Fitting independent datasets to the models resulted in relative error (*RE*, %) values of 12.6, 14.1, and 14.9% between estimated and measured aerial N uptake for mRPA, DIDA, and DDn across the range of −20° to +10°, respectively, further confirming the superior test performance of the mRPA index. These results illustrate that the novel index mRPA represents a more accurate assessment of plant N status, which is beneficial for guiding N management in winter wheat.

## Introduction

Nitrogen (N) is a vital element for higher photosynthetic functioning; N resource management is a major factor that can enhance plant growth and influence the quality of plant crops (Woodard and Bly, 1998; Smil, 2002). To ensure productivity, crop producers commonly supply plants in the field with N fertilizers. N supply generally appreciably surpasses plant N uptake, leading to the loss of nitrate through soil leaching, increased greenhouse gas (N_2_O) emission, and ground water pollution (Sehy et al., 2003; Ju et al., 2006). To minimize potential N losses, N fertilizer should be applied at the correct time and according to the requirements of the crops. The development, therefore, of techniques which insure higher yield and good product while reducing ecological environment pollution attributed to unsuitable N application is essential.

In currently, remote sensing technique is among the most promising approach that has been shown to rapidly predict the spatial-temporal variability of crops and monitor crop growth status (Hansen and Schjoerring, 2003; Ecarnot et al., 2013). Many indices have been constructed by extracting characteristic spectral information for evaluating biochemical properties in crop plants (Hatfield et al., 2008; Ecarnot et al., 2013). Several researchers have demonstrated a close relationship between NDVI- and RVI-like spectral indices and aboveground N uptake (Mistele and Schmidhalter, 2008; Li et al., 2013), chlorophylls and carotenoids (Blackburn, 1998), canopy leaf biomass (Le Maire et al., 2008). An additional group of vegetation indices are constructed by the forms of three band combination. For instance, Wang et al. (2012) added the 2 × R_423_ band to the NDVI (R_703_, R_924_) and effectively improved the sensitivity of leaf nitrogen concentration (LNC) estimation in rice and wheat. Feng et al. (2015) showed that the three-band spectral index (R_759_-1.8 × R_419_)/(R_742_-1.8 × R_419_) was a good indicator of above ground N uptake in wheat. A third group vegetation indices were developed by area-based algorithm. These include the triangle vegetation index (TVI) and modified TVI for green leaf area index (LAI) (Broge and Leblanc, 2000; Haboudane et al., 2004); the red-edge reflectance curve area for green biomass (Ren et al., 2011); and the adjusted TVI for aerial N uptake (Li et al., 2013). Additionally, remote sensing technology was also applied in the field of phenotype. Rothamsted Research reported that the data collecting from Unmanned Aerial Vehicle (UAV) based remote sensing could rapidly and accurately measure the wheat plant height and growth rate (Holman et al., 2016). Andradesanchez et al. (2014) investigated that the tractor-based phenotyping system could acquire and record data for canopy temperature, height and reflectance of cotton plants at much higher rates. However, the canopy spectral reflectance was sampled only from the vertical observation angle in prior researches, and the nadir observation were difficult to extract spatial structure from the middle and lower layers of plants (Thenkabail et al., 2000; Erdle et al., 2011).

Compared with the nadir observations, multi-angle observations contain more detailed and reliable canopy structure information that permits effective monitoring of crop N status in the middle and lower layers and provides a novel approach for quantitative remote sensing (Pocewicz et al., 2007; Huang et al., 2011). To date, many studies have shown that multi-angle measurements could improve the performance of indices when estimating the structural characteristics of ground objects (Shibayama and Wiegand, 1985; Diner et al., 1999). For instance, Galvão et al. (2009) showed that the varieties of soybean could be distinguished with the best predictive ability in the backward scattering direction. Gemmell and McDonald (2000) showed that the performance of indices under off-nadir angle can effectively discriminate forest cover and LAI. Furthermore, some studies have used to multi-angular datasets to assess plant variables, particularly biochemical components (Hasegawa et al., 2010; Huang et al., 2011). The effect of indices in estimating agronomic parameters and yields changes with the LAI and VZAs (Gemmell and McDonald, 2000; Inoue et al., 2008). Stagakis et al. (2010) focused on using satellite spectral data to estimate chlorphyll a (chl a), chl b, and carotenoids of semi-deciduous shrubs by utilizing different viewing angles and narrow-band indices. He et al. (2016) developed a multi-angular VI to enhance the estimation stability and accuracy of leaf nitrogen concentrations. No matter what the understory vegetation was (green or senesced) the relationships between canopy or total LAI and NDVI or Enhanced Vegetation Index (EVI) varied little across VZAs in pine forests (Pocewicz et al., 2007). However, only a few researches systematically tested the ability of multi-angle spectral data for predicting aerialN uptake of wheat. Taken together, these studies report the construction of a range of novel indices that use multi-angle datasets to enhance the precision and robustness of prediction indices for plant biophysical traits.

The specific aims of the paper were: to (1) study the performance of ground-based spectra and common indices to detect the aerial N uptake of winter wheat under different VZAs; (2) construct an improved, novel VI for aerial N uptake prediction; (3) compare the aerial N uptake predictive ability of the novel model with published VIs; and (4) establish the best observation angle and the best estimation model for aerial N uptake. The results of this study provide technical knowledge and a theoretical basis for monitoring N status by remote sensing technology; the information obtained from these techniques can then be used to help guide appropriate N fertilization application of wheat.

## Materials and methods

### Experimental fields

The field experiments were designed over a 4-year period in Zhengzhou and Shangshui city, China. The different locations, N fertilizer rates, wheat cultivars, and growth seasons were used (Table 1). Urea as N sources was divided into two equal doses, one administered before seeding and the rest at jointing period. Before seeding, 150 kg ha^−1^ P_2_O_5_ [as Ca(H_2_PO_4_)_2_] and 90 kg ha^−1^ K_2_O (as KCl) were used to all treatments. The N treatments with triplicates were assigned as completely random blocks in the experiment. The density of the seedlings was 3.0 × 10^6^ plants ha^−1^.

**Table 1:** The experimental conditions, N fertilizer levels, and measured stages.

**Exp. No**.	**Season and site**	**Cultivar**	**Soil characteristics**	**Treatments**	**Stages**
Exp. 1	2012–2013 Zhengzhou city	Yumai 49–198 Zhengmai9694	Type: fluvo-aquic soil, Organic-M: 17.47 kg^−1^, Soil pH (CaCl2): 7.9, Total N: 0.84 g kg^−1^, NO_3_-N: 8.1 mg kg^−1^, Available N:78.4 mg kg^−1^, Available P: 18.83 mg kg^−1^, Available K: 252.56 mg kg^−1^	N rate (kg ha^−1^): N0(0), N1(120), N2(240), N3(360), and 50% prior to seeding and 50% at jointing	Jointing Booting Heading Anthesis
Exp. 2	2013–2014 Zhengzhou city	Yumai 49–198 Zhengmai 9694	Type: fluvo-aquic soil, Organic-M: 16.8 g kg^−1^, Soil pH (CaCl2): 7.8, Total N: 0.89 g kg^−1^, NO_3_-N: 9.3 mg kg^−1^,Available N:113.0 mg kg^−1^, Available P: 19.20 mg kg^−1^, Available K: 252.30 mg kg^−1^	N rate (kg ha^−1^): N0(0), N1(120), N2(240), N3(360), and 50% prior to seeding and 50% at jointing	Jointing Booting Heading Anthesis
Exp. 3	2013–2014 Shangshui city	Zhoumai 27	Type: lime concretion black soil, Organic-M: 20.8 g kg^−1^, Soil pH (CaCl2): 7.1, Total N: 1.36 g kg^−1^, NO3-N:14.1 mg kg^−1^, Available N: 93.2 mg kg^−1^, Available P: 4.92 mg kg^−1^, Available K: 176.1 mg kg^−1^	N rate (kg ha^−1^): N0(0), N1(180), N2 (240), N3(300), and 50% prior to seeding and 50% at jointing	Jointing Booting Anthesis
Exp. 4	2014–2015 Zhengzhou city	Yumai 49–198 Zhengmai9694	Type: fluvo-aquic soil, Organic-M: 9.7 g kg^−1^, Soil pH (CaCl2): 8.01, Total N: 0.71 g kg^−1^ NO_3_-N: 7.2 mg kg^−1^, Available N: 64.6 mg kg^−1^, Available P: 28.8 mg kg^−1^, Available K: 101.7 mg kg^−1^	N rate (kg ha^−1^): N0(0), N1(120), N2 (240), N3(360), N4(450), and 50% prior to seeding and 50% at jointing	Jointing Booting Heading Anthesis
Exp. 5	2014–2015 Shangshui city	Yumai 49–198	Type: lime concretion black soil, Organic-M: 21.7 g kg^−1^, Soil pH (CaCl2): 8.06, Total N: 1.13 g kg^−1^, NO_3_-N: 10.6 mg kg^−1^, Available N: 85.7 mg kg^−1^, Available P: 13.1 mg kg^−1^, Available K: 111.3 mg kg^−1^	N rate (kg ha^−1^): N0(0), N1 (120), N2 (240), N3(360), and 50% prior to seeding and 50% at jointing	Jointing Booting Anthesis
Exp. 6	2011–2012 Zhengzhou city	Yumai 49–198 Zhengmai9694	Type: fluvo-aquic soil, Organic-M: 10.6 g kg^−1^, Soil pH (CaCl2): 7.9, Total N: 0.91 g kg^−1^, NO_3_-N: 8.4 mg kg^−1^, Available N: 82.0 mg kg^−1^, Available P: 25.6 mg kg^−1^, Available K: 124.5mg kg^−1^	N rate (kg ha^−1^): N0(0), N1(120), N2 (240), N3(360), and 50% prior to seeding and 50% at jointing	Booting Heading Anthesis Initial-fillingMid-filling

### Data acquisition

#### Measurement of canopy multi-angular hyperspectral reflectance

**Figure 1:**
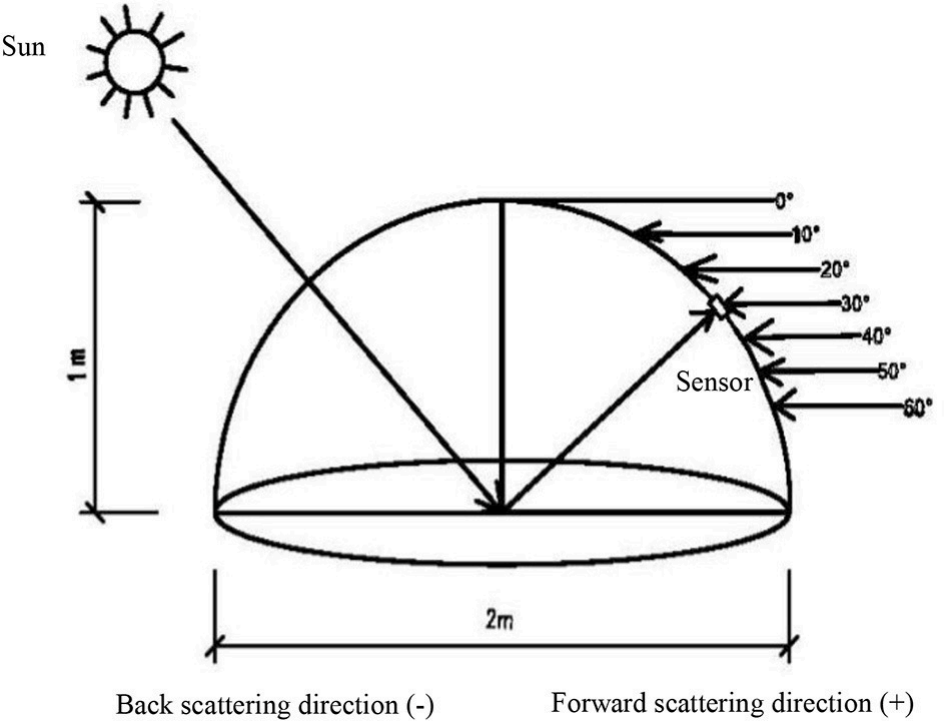
Dimensions and design of Field Goniometer System.

Canopy reflectance spectra were obtained in a 1 m^2^ area in each plot under sunny and windless conditions between11:00 to 13:00 using an ASD (Analytical Spectral Devices Inc., Boulder, CO, USA) FieldSpec Handheld spectrometer. This spectrum instrument was equipped with 25°-field-of-view optics fiber, sampling interval of 1.6 nm and spectral resolution of 3.5 nm from 325 to 1,075 nm. The multi-angle data are obtained with a Field Goniometer System, which was designed based on the system developed by Sandmeier and Itten (1999). The goniometer is a device used to position a sensor at these different angles and azimuths (Figure 1). The observed azimuth was fixed relative to the direction of the sun, and the measured plane was defined as the SPP (Myneni et al., 1995). The VZA was divided into backward direction (the observation direction same to the sun, −) and the forward direction (the observation direction against to the sun, +); the nadir position was defined as 0°. The VZA from backward to forward direction is −60, −50, −40, −30, −20, −10, 0, 10, 20, 30, 40, 50, 60°. The VZA become larger from 0° to ±60°, regardless of the backward and forward direction. The 10 sites were averaged to a single spectral sample of each plot. The black and base-line reflectance was calculated by a 40 × 40 cm BaSO_4_.

### Plant measurements

The areas of 0.20 m^2^ of plant samplings were randomly uprooted from each test area almost simultaneously with the canopy spectral acquisition. The plant samples were weighed after desiccation in an oven at 70°C to a constant weight. The ovendried plants were grinded into powder (1 mm) for N content analysis in laboratory. The aerial nitrogen concentration was determined in line with the micro-Kjeldahl method (Isaac and Johnson, 1976).

### Construction of the new VI

**Figure 2:**
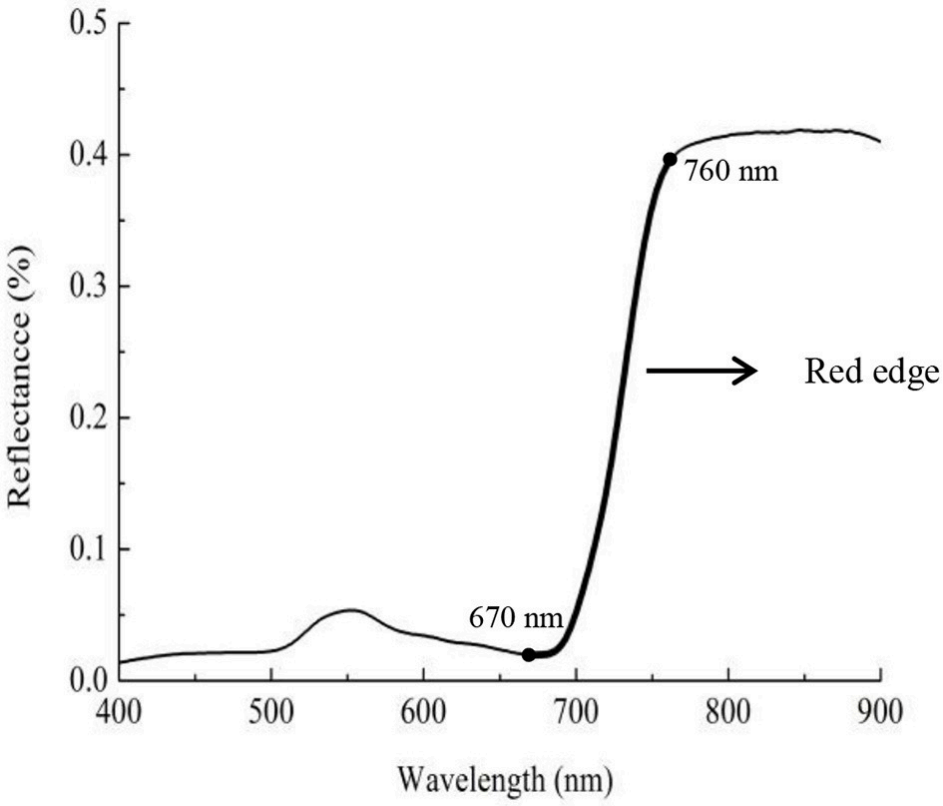
Schematic representation of red edge reflectance curve.

**Figure 3:**
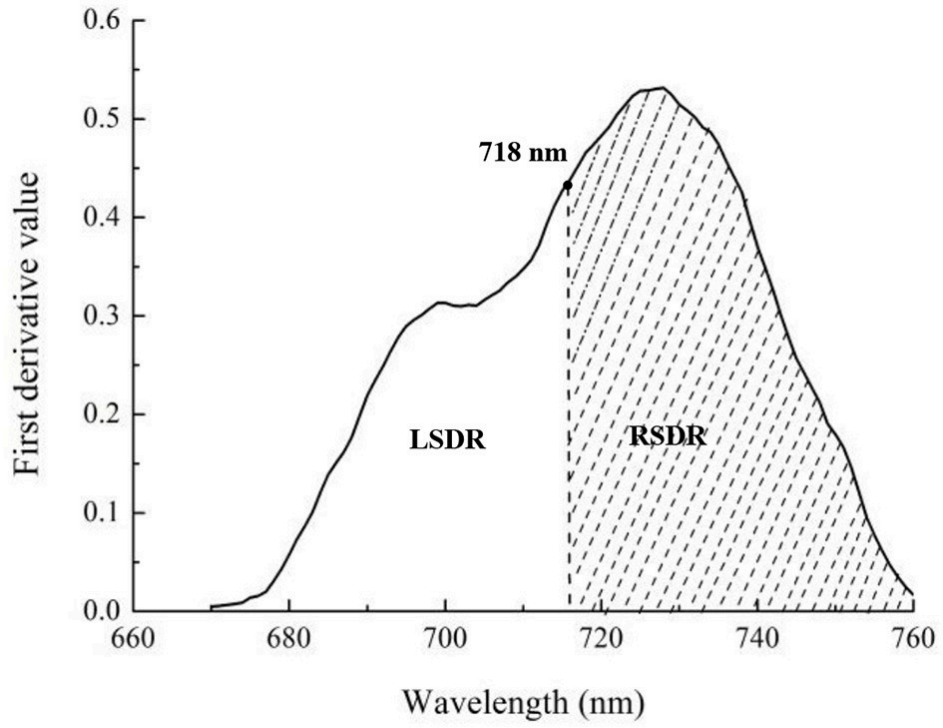
The division of the whole red-edge double-peak area into left and right single peak areas.

**Figure 4:**
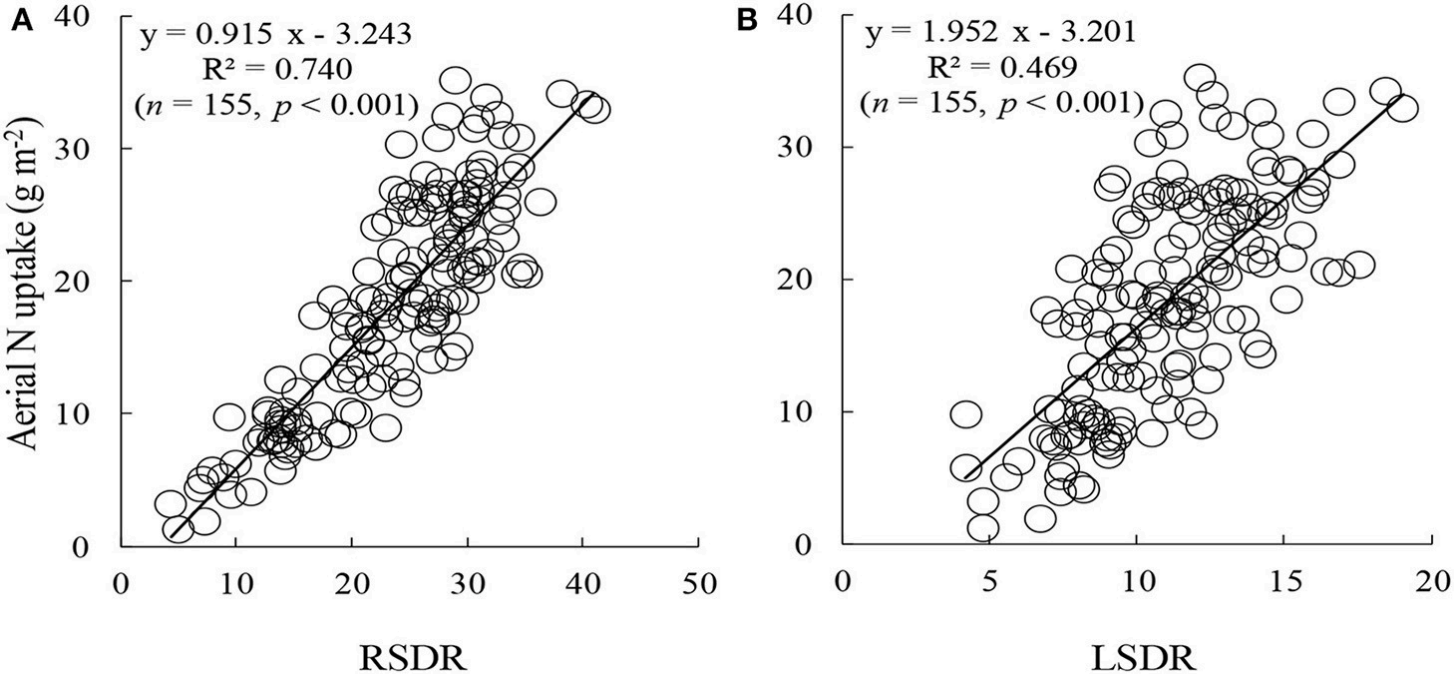
The relationships of aerial N uptake to RSDR **(A)** and LSDR **(B)** at 0°view zenith angle.

**Figure 5:**
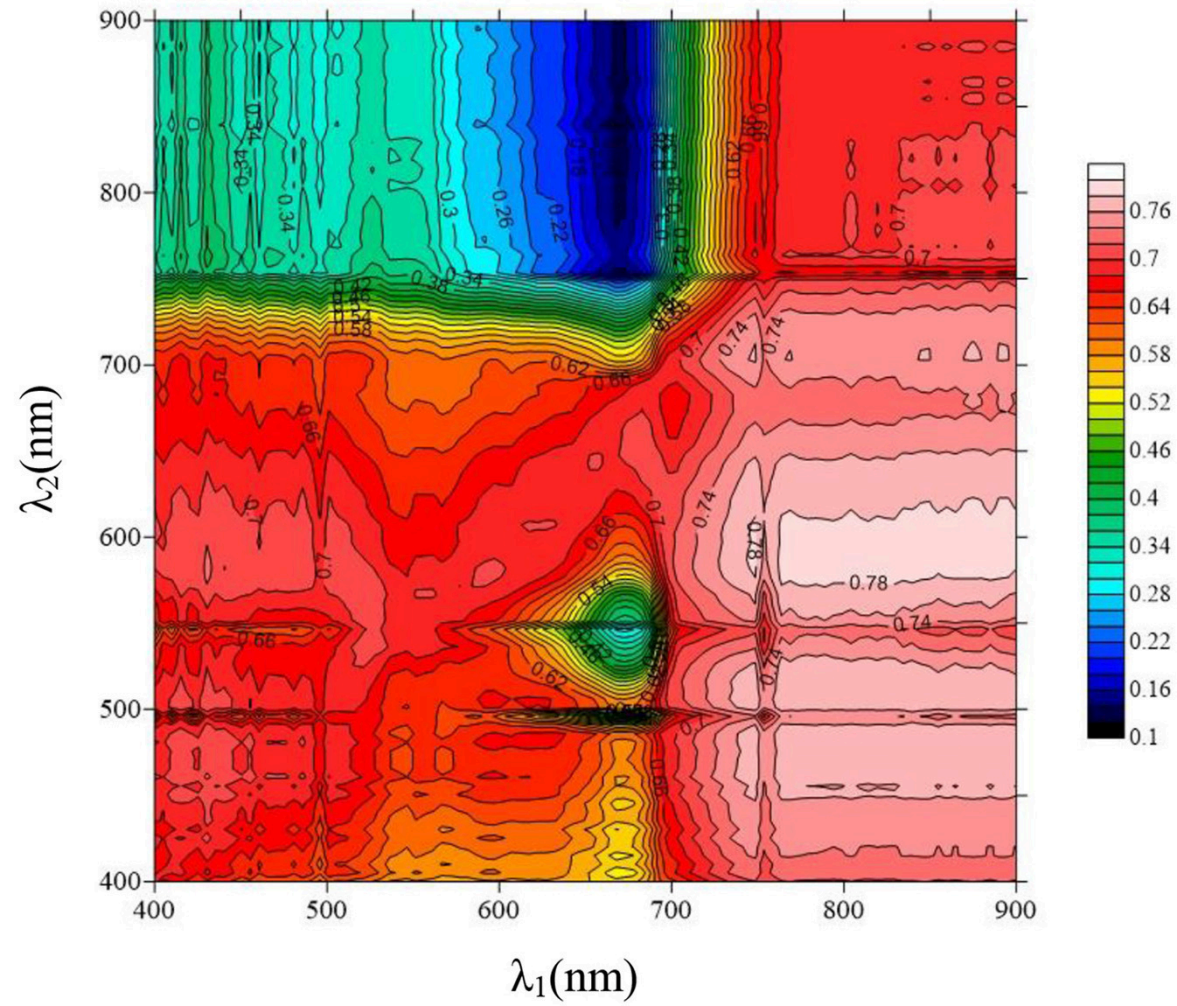
Contour maps of coefficients of determination between aerial N uptake and the mRPA with formula (R_λ1_/R_λ2_)^1/2^ × (R_760_-R_718_) (*n* = 155, *p*_0.001_ = 0.262).

The red edge is a region of steep variations in spectral reflectance, and this value may provide some useful information on crop growth and N status (Figure 2) (Sims and Gamon, 2002; Cho and Skidmore, 2006; Hatfield et al., 2008; Feng et al., 2015). To date, few area-based optimized indices have been constructed using red-edge information for non-destructive, rapid assessment of plant N status. Our preliminary research found that the double-peak area parameters constructed based on analysis of the red-edge double-peak characteristics could be effective for assessing Leaf N concentration (LNC) (Feng et al., 2014). There are several techniques to divide the red edge double-peak area into the right-side peak area (RSDR) and the left-side peak area (LSDR) (Figure 3). In this study, the datasets obtained using a 0° observation angle in Exp. 1–5 were used to analyze the relationship between LSDR, RSDR(from different splitting methods), and aerial N uptake. The results showed that RSDR (R_760_-R_718_) divided by characteristic wavelength method had the best performance (*R*^2^ = 0.740, *p* < 0.001; Figure 4), which suggested RSDR as potential indicator for estimating aerial N uptake. The previous research showed that the ratios of two or more bands (such as R_801_/R_670_, R_801_/R_550_) could increase sensitivity to crop physiological traits and reduce variation because of external influencial factors (Daughtry et al., 2000). Haboudane et al. (2008) inserted the (R_700_/R_670_)^1/2^ into the TVI formula to decrease the combined impacts of the soil background reflectance. We, therefore, inserted a coefficient [in the form of (λ_1_/λ_2_)^1/2^] into RSDR (R_760_-R_718_) to construct a novel VI called the mRPA. The spectral region of the above λ_1_ and λ_2_ were located within 400 and 900 nm. Figure 5 gave a comprehensive overview of the correlation coefficients for any two band combinations (λ_1_, λ_2_), and this is valid for selecting the sensitive bands of aerial N uptake. The wavebands λ_1_ and λ_2_ ranged between 750–900 and 550–650 nm, respectively. This area had the highest precision, with *R*^2^ values above 0.78, especially *R*^2^ performed best when λ_1_ = 760 nm, λ_2_ = 600 nm. Therefore, the final formula of mRPA was:

(1) $$\begin{array}{r} \text{mRPA}={\left({\text{R}}_{760}/R_{600}\right)}^{1/2}\times \left(R_{760}-R_{718}\right) \end{array}$$

### Model calibration and validation

The datasets from Exp. 1–5 were used to construct the evaluating models, and the dataset from Exp. 6 were used to validate these aerial N uptake evaluating models. The correlation of aerial N uptake and VIs was examined by MATLAB 7.0. In this study, 18 VIs were selected and summarized in Table 2. The quantitative relationship between the optimal VI and aerial N uptake could be established based on the highest *R*^2^. The model's behavior was evaluated by employing the *R*^2^, RMSE, and *RE* (%). Among the indices, those with the highest *R*^2^ and the lowest RMSE and *RE* were considered as the best. RMSE and *RE* were calculated from actual and predicted values of samples according to Equations (2) and (3), respectively:

(2) $$\begin{array}{r} RMSE=\sqrt{\frac{1}{n}\times \overset{n}{\underset{i=1}{\sum }}{\left(Pi-Oi\right)}^{2}} \end{array}$$

(3) $$\begin{array}{r} RE\left(\%\right)=\sqrt{\frac{1}{n}\times \overset{n}{\underset{i=1}{\sum }}{\left(\frac{Pi-Oi}{Oi}\right)}^{2}}\times 100\% \end{array}$$

Here, *P*_i_, and *O*_i_ represented the estimated and measured values, respectively, and *n* represented the sampling number. The prediction was considered to be excellent if *RE* < 10%, good if *RE* = 10–20%, fair if *RE* = 20–30%, and poor if *RE* > 30% (Feng et al., 2014).

**Table 2:** Summary of selected vegetation indices published in the literature.

**Spectral index**	**Definition or equation**	**Reference**
**TWO BANDS**		
**Optimal vegetation index (VIopt)**	**(1+0.45) × [(R_800_)^2^+1)/(R_670_+0.45)]**	**Reyniers et al., 2006**
**Difference vegetation index (DVI)**	**R_810_−R_560_**	**Richardson and Wiegand, 1977**
**Re-normalized difference vegetation index (RDVI)**	**(R_800_-R_670_)/(R_800_+R_670_)^1/2^**	**Roujean and Breon, 1995**
**Pigment specific simple ratio chlorophyll b (PSSRb)**	**R_800_/R_635_**	**Blackburn, 1998**
**Modified simple ratio (MSR)**	**[R_800_/R_670_-1]/[(R_800_/R_670_)^1/2^ +1]**	**Chen, 2014**
**Normalized difference red-edge index (NDRE)**	**(R_790_-R_720_)/(R_790_+R_720_)**	**Fitzgerald et al., 2006**
**Soil adjusted vegetation index (SAVI)**	**(1-0.08) × (R_825_-R_735_)/(R_825_+ R_735_-0.08)**	**Huete, 1988**
**Red-edge chlorophyll index-3 (CIred-edge3)**	**R_790_/R_720_−1**	**Gitelson et al., 2005**
**THREE BANDS**		
**Difference index of the double-peak areas (DIDA)**	**(R_755_+R_680_-2 × R_718_)/(R_755_-R_680_)**	**Feng et al., 2014**
**New double difference index (DDn)**	**2 × R_710_-R_660_-R_760_**	**Le Maire et al., 2008**
**Modified chlorophyll absorption in reflectance index (MCARI-1)**	**1.2 × [(2.5 × (R_800_-R_670_)−1.3 × (R_800_-R_550_)]**	**Haboudane et al., 2004**
**Modified triangular vegetation index (MTVI1)**	**1.2 × [1.2 × (R_800_-R_550_)−2.5 × (R_670_-R_550_)]**	**Haboudane et al., 2004**
**Modified red-edge ratio (mRER)**	**(R_759_−1.8 × R_419_)/(R_742_−1.8 × R_419_)**	**Feng et al., 2015**
**Modified red-edge ratio (mSR705)**	**(R_750_-R_455_)/(R_705_-R_445_)**	**Sims and Gamon, 2002**
**Modified right-side peak area index (mRPA)**	**(R_760_/R_600_)^1/2^×(R_760_-R_718_)**	**This study**
**OVER THREE BANDS**		
**SDr-SDb**	**$\int_{680}^{760}\frac{dR\lambda }{d\lambda }d\lambda -\int_{490}^{530}\frac{dR\lambda }{d\lambda }d\lambda$**	**Feng et al., 2008**
**Triangle vegetation index (TVI-3)**	**60 × (R_nir_-R_green_)−100 × (R_red_-R_green_)**	**Broge and Leblanc, 2000**
**Red-edge reflectance curve area (REFCA)**	**SUM(R_i_/R_780_) i = 680–780**	**Ren et al., 2011**

*R is the reflectance at a given wavelength. R_800_, R_670_, R_635_,…and R_680_ are the spectral reflectance values at 800, 670, 635…, and 680 nm, respectively. R_λ_ is the spectral reflectance at wavelength λ*.

## Results

### Variability of wheat aerial N uptake under different growth stages

**Figure 6:**
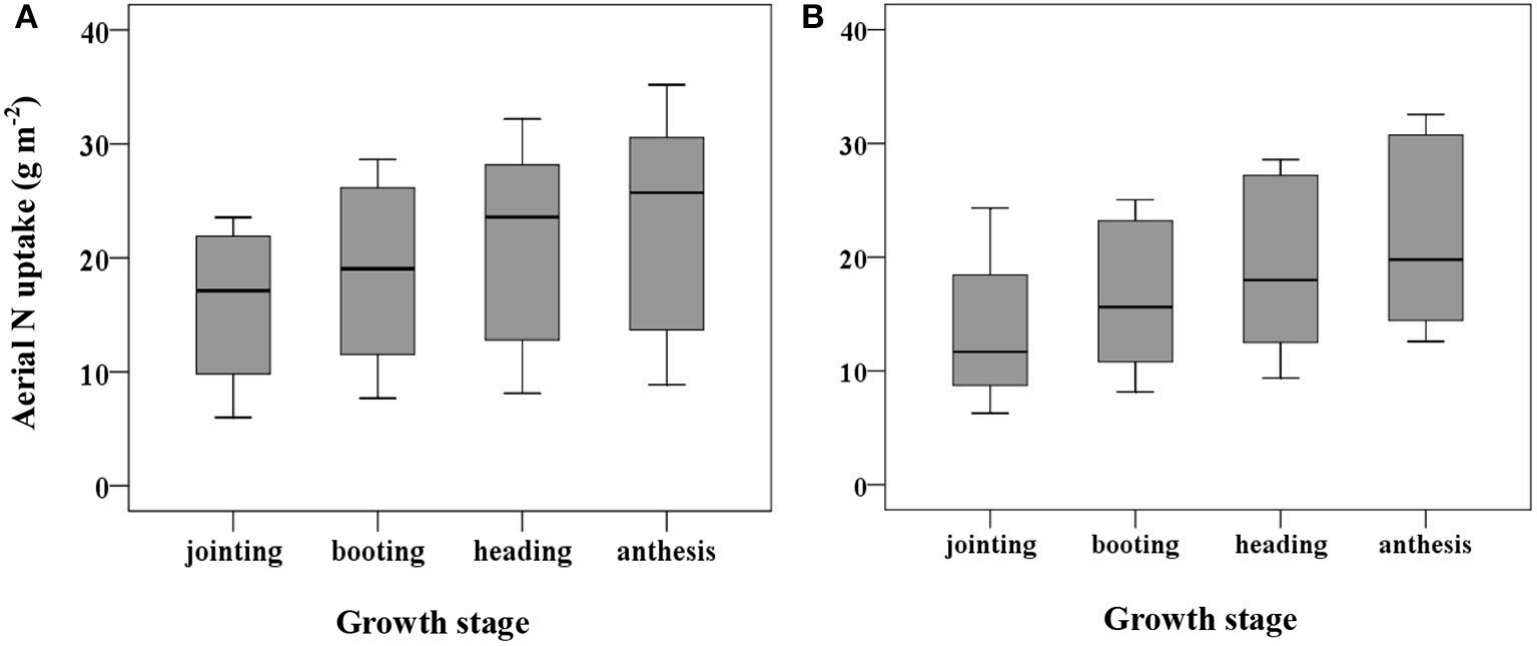
Variation in plant N uptake over jointing-anthesis growth stages in wheat cultivars of Yumai 49-198 **(A)** and Zhengmai 9694 **(B)**.

The datasets from Exp. 1 are shown in Figure 6 to illustrate the general distribution of aerial N uptake. The aerial N uptake of the two wheat cultivars increased in the vegetative period because of increasing biomass. Across the different applied N rates, the aerial N uptake of Yumai 49–198 ranged from 6.0–23.5, 7.7–28.6, 8.1–32.2, and 8.9–35.1 g kg^−1^ in the jointing, booting, heading, and anthesis stage, respectively. The aerial N uptake of Zhengmai 9694 varied from 6.9–24.3, 9.3–25.1, 10.6–27.1, and 13.4–31.2 g kg^−1^, respectively, in these stages. With the progression of the growth stages, coefficients of variation for aerial N uptake increased. Thus, it can be seen that the aerial N uptake was significantly influenced by the different wheat cultivars and growth period.

### Relationship between canopy reflectance and aerial N uptake at different VZAs

**Figure 7:**
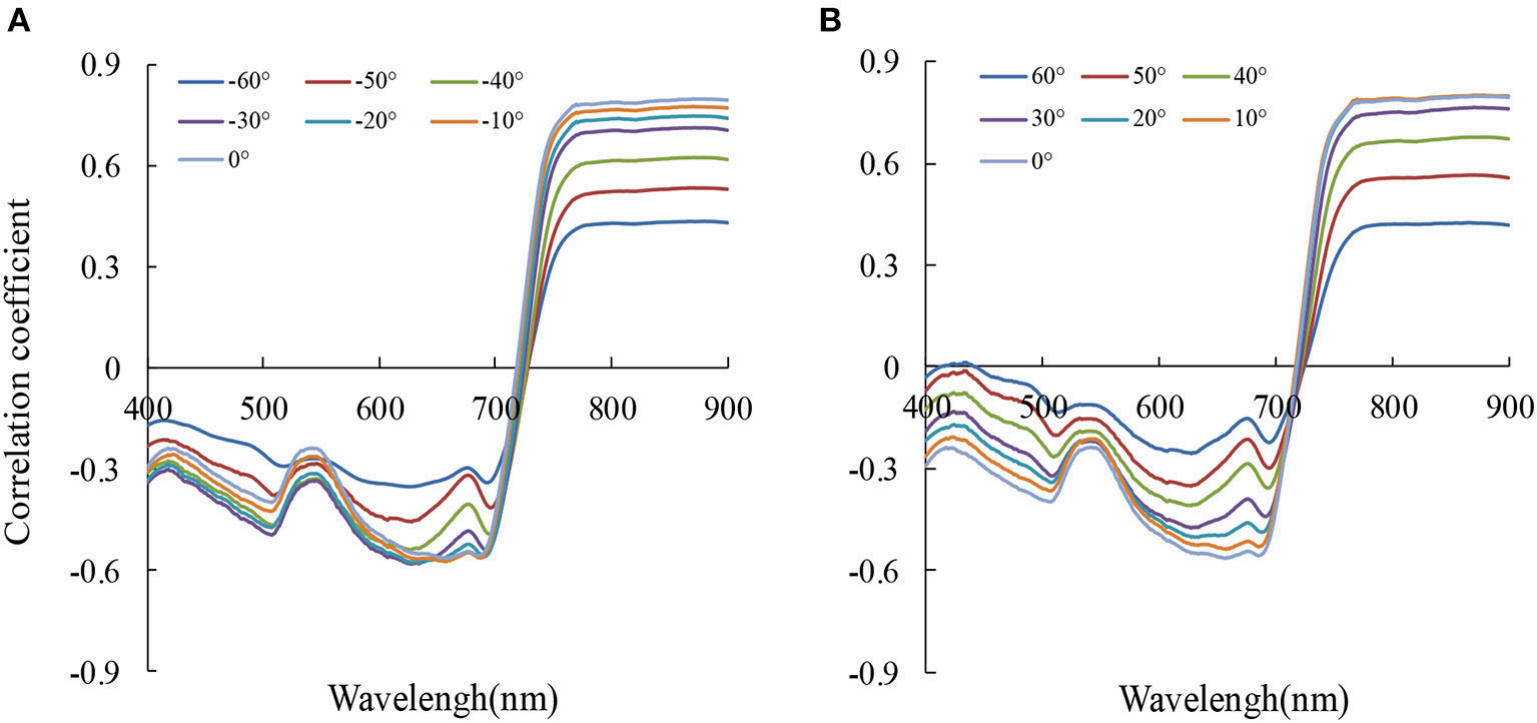
The correlation coefficient (*r*) between reflectance (*R*_*x*_) and aerial N uptake at 13 viewing zenith angles (**A**: backward scattering, **B**: forward scattering) (*n* = 155, *p*_0.001_ = 0.262).

We plotted the correlation between canopy reflectance and aerial N uptake under different VZAs with the data from Exp. 1–5 (Figure 7). In the 13 VZAs, a negative relationship was detected between aerial N uptake and the reflectance in the 400–720 nm. The minimum correlation coefficient was under 560–710 nm (r < −0.57), caused by red valley and chlorophyll absorption. The highest *r*-value was presented in the near infrared region (NIR), and increased with decreasing VZA both in backward and forward scattering. No matter in the backward and forward scattering, the r sharply changed in the red band region (690–760 nm), and it tended to 0 near 720 nm; this indicates that *r* from this region was not sensitive to the VZA.

### Relationship between the aerial N uptake and spectral indices at different VZAs

Canopy spectral data of different VZAs were influenced by numerous comprehensive factors including soil background, meteorological conditions, leaves, stems, and spectral noise. In this study, 18 spectral indices, including the new index and 17 published indices are presented in Table 2. Among them, the common indices were chose in accordance with a comprehensive literature investigation of red edge characteristics. To further describe the ability of the spectral indices in assessing aerial N uptake, we used the datasets from Exp. 1–5 to compare the predictive ability. As shown in Table 3 the r from backward scattering directions were higher than forward scattering directions for most two-band indices (except for DVI and RDVI). To the performance of 13 single VZAs, the relatively high correlation coefficient within −40° to +30° was present. For the well-performance VIs, the mRPA, DIDA, and DDn were advantageous at a viewing angle of −10°, with r scores of 0.896, −0.877, and −0.872, respectively;CI_red−edge3_ and mSR705 were most sensitive at the −20° viewing angle, with r scores of 0.776 and 0.771, respectively; PSSRb and MSR demonstrated the best performance at the 0° viewing angle, with r scores of 0.808 and 0.789, respectively; SDr-SDb and TVI-3 had the higher correlations at the +10° viewing angle, with r scores of 0.814 and 0.809, respectively. Notably, eight of 18 indices produced the best correlations at the −10° viewing angle. These results illustrate that the around −10° VZA may be the most suitable for aerial N uptake estimation.

**Table 3:** The correlation coefficient (*r*) for the relationships of vegetation indices with aerial N uptake at different viewing zenith angles (*n* = 155, *p*_0.001_ = 0.262).

	**−60°**	**−50°**	**−40°**	**−30°**	**−20°**	**−10°**	**0°**	**10°**	**20°**	**30°**	**40°**	**50°**	**60°**
**TWO BANDS**													
VIopt	0.560	0.644	0.732	0.795	0.831	0.858	0.855	0.829	0.764	0.712	0.643	0.531	0.390
DVI (810,560)	0.548	0.627	0.711	0.779	0.806	0.832	0.828	0.823	0.811	0.791	0.719	0.624	0.493
RDVI (800,670)	0.535	0.591	0.672	0.743	0.781	0.827	0.825	0.808	0.803	0.763	0.694	0.599	0.475
PSSRb	0.490	0.641	0.715	0.765	0.795	0.802	0.808	0.753	0.710	0.679	0.624	0.539	0.412
MSR	0.460	0.571	0.655	0.718	0.761	0.787	0.789	0.752	0.685	0.627	0.540	0.423	0.306
NDRE	0.602	0.683	0.732	0.759	0.774	0.778	0.779	0.760	0.738	0.715	0.689	0.646	0.583
SAVI (825,735)	0.565	0.660	0.706	0.742	0.765	0.764	0.758	0.749	0.734	0.715	0.687	0.643	0.587
CIred-edge3	0.558	0.670	0.726	0.758	0.776	0.766	0.753	0.748	0.728	0.702	0.674	0.626	0.552
**THREE BANDS**													
DIDA	−0.699	−0.736	−0.792	−0.828	−0.853	−0.877	−0.871	−0.869	−0.842	−0.818	−0.789	−0.752	−0.672
DDn	−0.656	−0.715	−0.786	−0.827	−0.850	−0.872	−0.867	−0.865	−0.838	−0.812	−0.784	−0.724	−0.614
MTVI1	0.411	0.484	0.577	0.671	0.722	0.756	0.781	0.785	0.771	0.724	0.635	0.519	0.387
mRER	0.592	0.601	0.603	0.632	0.705	0.780	0.777	0.775	0.712	0.679	0.674	0.616	0.558
mSR705	0.504	0.643	0.719	0.753	0.771	0.769	0.749	0.713	0.674	0.653	0.612	0.534	0.407
mRPA	0.653	0.738	0.816	0.857	0.879	0.896	0.893	0.880	0.853	0.823	0.783	0.711	0.578
**OVER 3 BANDS**													
DD	0.656	0.689	0.759	0.807	0.835	0.868	0.862	0.857	0.847	0.821	0.772	0.701	0.588
SDr-SDb	0.486	0.573	0.661	0.740	0.775	0.787	0.798	0.814	0.802	0.762	0.678	0.570	0.434
TVI-3	0.457	0.546	0.637	0.723	0.761	0.789	0.808	0.809	0.800	0.761	0.676	0.565	0.426
REFCA	−0.609	−0.655	−0.703	−0.736	−0.754	−0.750	−0.748	−0.741	−0.704	−0.695	−0.661	−0.621	−0.566

**Figure 8:**
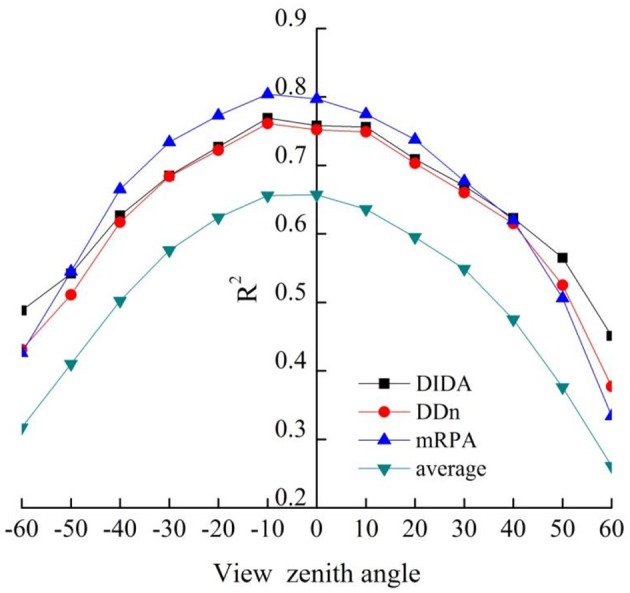
Relationship between aerial N uptake and DIDA, DDn, mRPA, and average (corresponding average value of 18 VIs at different VZAs, shown as average) at different VZAs (*n* = 155, *p*_0.001_ = 0.262).

The four indices [mRPA, the two best-performing common indices (DIDA and DDn, and average (corresponding average value of 18 VIs at different VZAs, shown as average)] based on the *R*^2^ values of the correlations between VIs and aerial N uptake were plotted in Figure 8. The results demonstrated that *R*^2^ increased with decreasing VZA in both backward and forward scattering directions, and the highest *R*^2^ were obtained under −20° to +10° VZAs. DIDA, DDn, and mRPA had strong correlations (*R*^2^ > 0.72) to aerial N uptake in this region. Nevertheless, the average did not show any strong correlations with aerial N uptake (*R*^2^ < 0.66). Compared with the average from −20° to +10° VZAs, the *R*^2^ of mRPA, DIDA, and DDn was increased by 21.3–23.9, 15.3–18.8, and 14.4–17.6%, respectively.

### Suitable combined angles for aerial N uptake assessment using VIs

**Figure 9:**
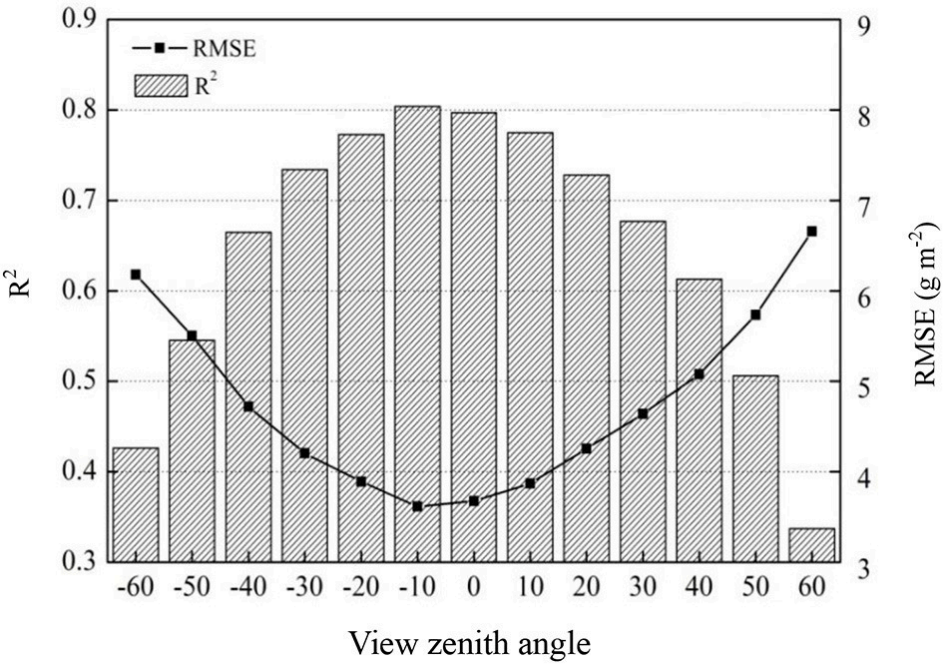
Comparison of the prediction power of mRPA at 13 VZAs in terms of aerial N uptake (*n* = 155, *p*_0.001_ = 0.262).

**Figure 10:**
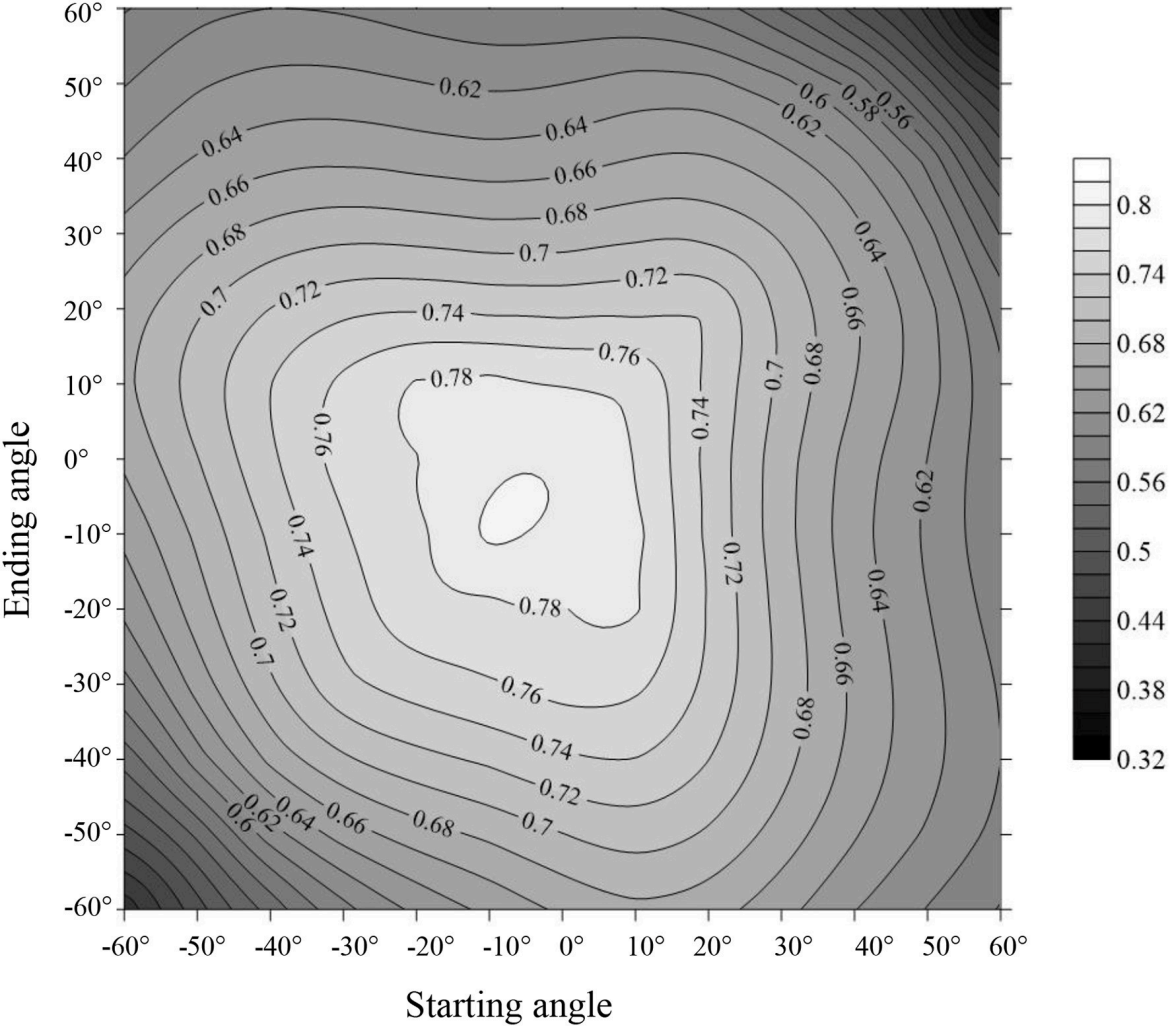
Comparison of the predictive ability (*R*^2^) of the indices within different view zenith angles combinations in terms of aerial N uptake.

**Figure 11:**
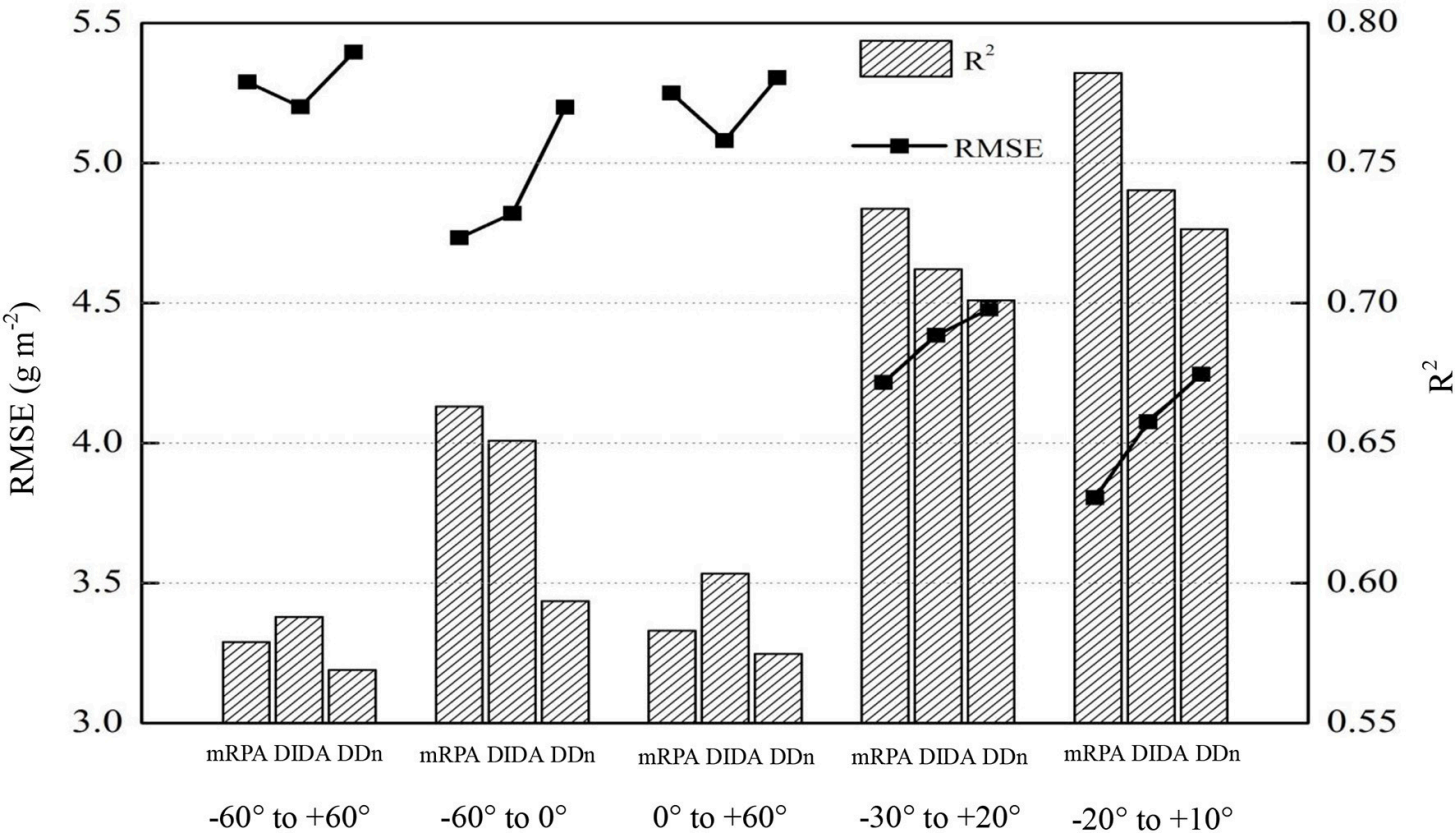
Comparison of the predictive ability of the indices within five kinds of view zenith angles combinations in terms of aerial N uptake.

**Figure 12:**
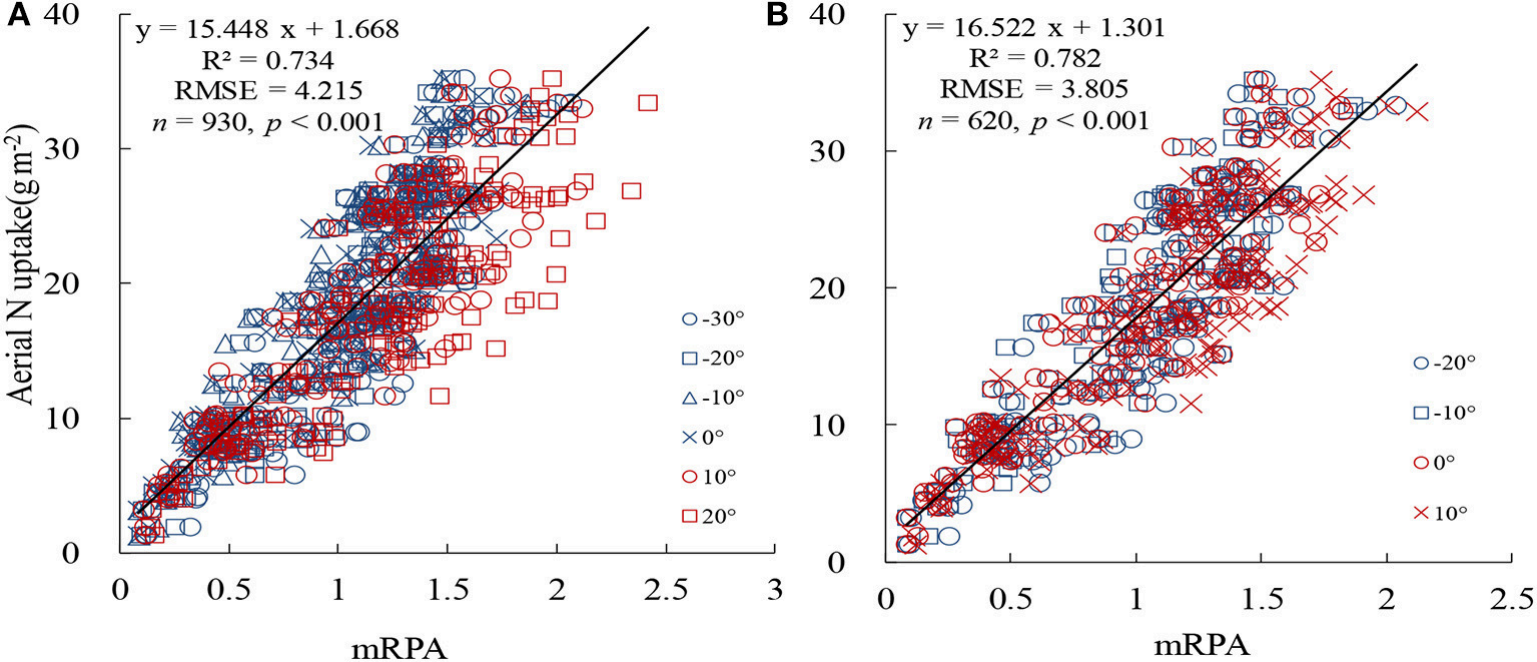
Comparison of the prediction power of mRPA at different VZA combinations in terms of aerial N uptake. **(A)** −30° to +20° and **(B)** −20° to +10°.

To ascertain the suitable range of VIs to the mRPA, *R*^2^ and RMSE were selected to compare different angle combinations. As showed in Figure 9, the performance of the *R*^2^ and RMSE in the back scattering direction (*R*^2^ = 0.426–0.804, *p* < 0.001) were superior to those in the forward view angles (*R*^2^ = 0.337–0.775, *p* < 0.001). Among the 13 VZAs, −20° to +10° VZAs showed significantly higher predictive ability (high *R*^2^ and small RMSE), and the most significant viewing angle was found to be −10°, with an *R*^2^ and RMSE of 0.804 and 3.615, respectively. Figure 10 revealed that these combinations including large VZAs combinations generated relatively poor correlations. There was a dominant region in range of −10° to 0° (*R*^2^ = 0.796), and the predictive ability of mRPA in −20° to +10° combination was also relatively high (*R*^2^ = 0.782). When the VZA was out of −40° to +20°, the predictive accuracy was relatively low (*R*^2^ < 0.740). A comparison of VIs (Figure 11) demonstrated that the mRPA (*R*^2^ = 0.782 and 0.734) at −20° to +10° and −30° to +20° VZAs were more sensitive than the two best-performing published index DIDA (*R*^2^ = 0.740 and 0.712) and DDn (*R*^2^ = 0.726 and 0.701). Figure 12 showed the quantitative relationship between aerial N uptake and mRPA. The *R*^2^ increased from 0.734 (from −30° to +20° combination) to 0.782 (from −20° to +10° combination). Compared to the −10° VZA having the highest *R*^2^ value, mRPA in −20° to +10° combination only had a slightly decreased *R*^2^ (2.7%) and an increased RMSE (5.2%). As a result, the novel mRPA model is the most forceful index for assessing aerial N uptake because of its insensitivity to VZAs of −20° to +10°, increasing the practicality of mRPA in actual production process.

### Testing aerial N uptake estimation models

**Figure 13:**
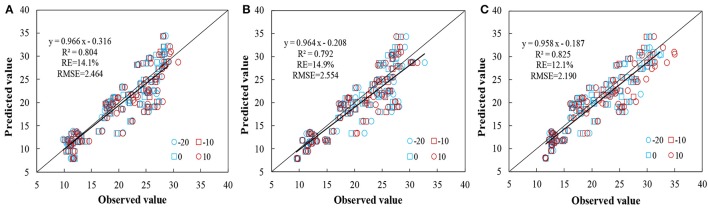
Comparison between estimated and measured aerial N uptake based on DIDA **(A)**, DDn **(B)**, and mRPA **(C)** for −20° to +10° combinations (*n* = 252, *p* < 0.001).

The relationship between aerial N uptake and the spectral indices (across −20° to +10° VZAs) discussed above were measured utilizing data from Exp. 6 using *R*^2^, RMSE, and *RE* to evaluate the accuracy and applicability between observed and predicted values. Data analysis was carried out on the common best-performing VIs DIDA and DDn, and on the novel index mRPA (Figure 13). The DIDA and DDn showed acceptable performance in the tests, with *R*^2^ of 0.804, *RE* of 14.1%, and a RMSE of 2.464 for DIDA and *R*^2^ of 0.792, *RE* of 14.9%, and a RMSE of 2.554 for DDn; this also indicates that DIDA is a better indicator than DDn. Compared with these two common indices, the mRPA demonstrated the superior predictive ability of aerial N uptake, with *R*^2^ of 0.825, *RE* of 12.1% and RMSE of 2.190. In summary, mRPA seems to be the best index for predicting aerial N uptake of winter wheat under different management conditions.

## Discussion

Remote sensing technology is widely used in agricultural production. It mainly includes the following aspects: crop growth measuring (e.g., biomass, N content, and yield), agricultural disaster monitoring (e.g., plant diseases and insect pests, droughts and floods) and crop phenotyping (e.g., crop height, leaf size, shape, and canopy longevity), and so on (Le Maire et al., 2008; Cao et al., 2015; He et al., 2016; Holman et al., 2016; Virlet et al., 2017), which could provide technical support for production management. However, spectral reflectance has previously been shown to be significantly affected by canopy structure, planting density, the wind, the angle of the sun, and various other factors (Rondeaux et al., 1996). To extract exact information for different characteristics and improve the detection precision, area calculations have been introduced to reduce the background effects (Broge and Leblanc, 2000; Ren et al., 2011; Li et al., 2013). Delegido et al. (2010) developed the normalized area over reflectance curve index (NAOC) and this index showed a linear relation with chlorophyll content. The normalized difference of the double-peak areas (NDDA) was calculated and discovered to correlate strongly with LNC (Feng et al., 2014). Broge and Leblanc (2000) found that the TVI (constructed by the area under the concave curve of red light absorption) could effectively assess the chlorophyll content and LAI. The chlorophyll absorption integral infers chlorophyll concentration through calculating the surrounding area between a connecting line of 600 and 735 nm and the red edge (Oppelt and Mauser, 2004). In this study, five of 17 VIs have previously been constructed using the area-based algorithm. Among them, DIDA, SDr-SDb, and TVI had significant correlations with aerial N uptake, with *r*-values of −0.877, 0.814, and 0.809, respectively, at their advantageous viewing angles (−10, 10, and 10°, respectively). The best-performing common index DIDA had strong correlations (*R*^2^ > 0.74) to aerial N uptake within −20° to +10° VZAs. In addition, DIDA showed acceptable performance in the tests, with *R*^2^ of 0.804, *RE* of 14.1% and a RMSE of 2.464. The above results showed that the VIs constructed by area algorithm could potentially be used to precisely predict aerial N uptake.

Many vegetation indices have been also constructed, including those NDVI-, RVI-, and DVI-like spectral indices, or other derived functions to enhance accuracy of estimating models (Huete, 1988; Wang et al., 2012). Baret and Guyot (1991) and Rondeaux et al. (1996) constructed the transformed soil-adjusted vegetation index (TSAVI) and optimized SAVI (OSAVI) by adding soil line parameters into NDVI to decrease the sensitivity of the soil background reflectance at low LAI. In order to reduce the combined influences of the canopy non-photosynthetic materials and increase the sensitivity of chlorophyll concentration determination, Daughtry et al. (2000) added the R_700_/R_670_ to the chlorophyll absorption ratio index (CARI) to obtain modified CARI. Haboudane et al. (2008) brought the (R_700_/R_670_)^1/2^ into the TVI to construct the triangular chlorophyll index (TCI) to increase its sensitivity of chlorophyll changes. These studies inspired us to develop a novel index by adding a coefficient. We attempted to derive the coefficient by combining different bands in a square root form. Finally, R_760_ and R_600_ were selected from different combinations of bands and changed into the form of (R_760_/R_600_)^1/2^. This was integrated with RSDR (760,718) to construct the mRPA, and this novel VI had a high correlation coefficient within −20° to +10° VZAs (*R*^2^ = 0.782).The mRPA makes the best of area algorithms and red-edge information, and effectively improves the monitoring accuracy of aerial N uptake.

The vegetation indices displayed anisotropy depending on the canopy structural development, shadowing, the view angles of the sensors, the inherent viewing geometry of sensors, and in some respects the underlying soil (Kimes et al., 1985). Vegetation indices merging into multiple VZAs had the potency to further enhance anisotropy estimation. Pocewicz et al. (2007) took full advantage of the hotspot effect in the backscatter direction to improve quantitative estimation of LAI. Galvão et al. (2009) highlighted that the back-scattering direction was suited to predict the yield of soybean. He et al. (2016) showed that the novel VI constructed by a four-band VI from two angles (−20° and +10°) was sensitive to the change of the LNC in wheat. In this study, the back scattering direction improved indices performance for aerial N uptake compared with the forward scattering direction. The main reason may be that back scattering observations contain more signals from sunlit branches or leaves with higher reflectance values, while forward-scatter observations derive mostly from shady branches/leaves with lower reflectance values (Stagakis et al., 2010). In addition, our results showed that the *R*^2^ increased with decreasing view zenith angles in back and forward-scatter direction. This was mainly because that the spectral data obtained at small angles mainly includes the total plant characteristics (lower, middle, and upper) of wheat. In general, sampling involving upper, middle, and lower wheat leaf layers could determine the aerial N uptake of the target region. In summary, further survey analysis on the variations of relationships between canopy attributes and remote sensing observations and on the availability off-nadir in fetching information is recommended.

Multi-angular remote sensing is able to obtain three-dimensional vegetation structure information, and thus it is better than vertical measurement for monitoring the canopy structural properties and the biochemical component of ground objects (Pocewicz et al., 2007). Rautiainen et al. (2008) demonstrated that high VZAs are the best fit for detecting over-story LAI values because of the quite limited influence of bottom layer on the whole signal. The value of canopy chlorophyll inversion index(CCII) at ±50 and ±60°, ±30 and ±40°, and nadir, ±20 and ±30°, VZAs were selected for inverting the chlorophyll at the upright upper, middle and bottom layer (Huang et al., 2011). Song et al. (2016) showed that −40° VZA was suitable to effectively monitor the LNC of wheat. This study showed that the *R*^2^ of mRPA changed strongly with VZAs, which the highest *R*^2^ value was found at −10° VZA (*R*^2^ = 0.804). The mRPA within −10° could improve the monitoring accuracy of aerial N uptake. However, it is not convenient to accurately control measuring angle under off-nadir conditions in the field operation. Our research found that mRPA was relatively insensitive between −20° and +10° VZAs region for *R*^2^ changes. A comparison among spectral parameters demonstrated that performance of mRPA in −20° to +10° combined dataset was superior to the two better published indices, with *R*^2^ of 0.782 for mRPA, 0.750 for DIDA, and 0.736 for DDn, respectively. Compared to the most sensitive VZA (−10°), mRPA within −20° to +10° VZAs only had a slightly reduced *R*^2^ (2.7%). This allowed us to construct a unified model in variable VZAs range to assess aerial N uptake in wheat, which decrease the influence of the VZAs and increase the field operation simplicity and applicability in a wide-angle region using portable monitors. Therefore, it is vital to select appropriate VI formulas and VZAs, which could reduce the variance due to soil background and crop canopy structure. In summary, the novel index plays an important role in predicting aerial N uptake of wheat and could be utilized to more precisely regulate N fertilization rate for different cultivation sites and plant types.

In this study, the novel index mRPA had the higher predictive ability with range of −20° to +10°. This can not only provide optimized parameters for the development of the portable monitor, but also offer dynamic information for guiding precise N budgeting. In order to obtain higher yield and avoid wasting resources, it is important to consider crop-N demand as well as soil-N supply to optimum N fertilizer strategy (Ju et al., 2009; Hartmann et al., 2015). The N fertilizer requirement was calculated using the following formula: N_req_ = (N_target_ – N_uptake_ – N_soil_)/*f*_NUE_, where N_target_ is the total crop-N demand for a target yield and grain protein, calculated according to Angus (2001), N_uptake_ is the aerial N uptake, N_soil_ is the potential soil-N supply for the rest of the growing period, and *f*_NUE_ is the fertilizer-N use efficiency. The mRPA model developed in this study could effectively estimate N_uptake_, which will contribute to managing the N application in winter wheat. The prediction power of VIs was affected by cultivation factors. Only if the VI was seldom influenced by the factors of cultivation, the applicability of the model was strong. We synthesized dataset from the vegetative growth stages to develop a unified model that could be easily used to assess the N status. However, this research was designed only on winter wheat in Henan province, the dependability and adaptability of this novel model ought to be tested in other crops and areas.

## Conclusions

Timely assessment of aerial N uptake is important to diagnose crop N status, maximizing yields and minimizing disadvantageous environmental impacts. In this study, we compared the use of 18 VIs, including 17 common VIs and a novel index constructed in this study, to estimate aerial N uptake of wheat. The results demonstrated that back scattering observation angles improved the ability to predict aerial N uptake compared with forward-scatter viewing angles VZAs. To decrease the restrictions on the environmental conditions and to further explore the superiority of spectral information, we combined the advantages of red-edge characteristics and area-based algorithms to construct a novel index mRPA to illuminate dynamic changes in aerial N uptake. The novel VI have the characteristic of simplicity and reliability and could be developed according to the formula: mRPA = (R_760_/R_600_)^1/2^ × (R_760_-R_718_). Compared with the best-performing traditional indices DIDA and DDn, the predictive ability of mRPA at −10° view angle was effectively enhanced by 4.6–5.8%. Further systematic analysis of VZA combinations showed that mRPA had the best forecasting ability when compared with the traditional indices, with small difference between combinations of −20° to +10°. This has guided us in the development of a unified model for forecasting the aerial N uptake of wheat across a wide angle range; this will increase the precision of N predictions under a range of angles using portable monitors. The integrated index mRPA was shown to be practical and exact for aerial N uptake evaluation of winter wheat. This result will be also beneficial for choosing appropriate VZA and for the construction of more precise sensors for ecosystem monitoring. Nonetheless, it is also need to further validate the reliability and stability of the novel VI and to examine its effectiveness under the condition of different cultivation environment.

## Author contributions

B-BG, WF, and Y-JZ conceived the research. B-BG, Y-PW, YZ, and X-XR performed the experiments. B-BG and WF wrote the paper. LH and YM contributed to the results analysis and discussion.

### Conflict of interest statement

The authors declare that the research was conducted in the absence of any commercial or financial relationships that could be construed as a potential conflict of interest.

## Abbreviations

ASD: Analytical Spectral Devices
CARI: Chlorophyll absorption ratio index
Chl a: Chlorphyll a
CIred-edge3: Red-edge chlorophyll index-3
DDn: New double difference index
DIDA: Double-peak areas
DVI: Difference vegetation index
EVI: Enhanced Vegetation Index
LAI: Leaf area index
LNC: Leaf N concentration
LSDR: Left-side peak area
MCARI-1: Modified chlorophyll absorption in reflectance index
mRER: Modified red-edge ratio
mRPA: Modified right-side peak area index
MSR: Modified simple ratio
mSR705: Modified red-edge ratio
MTVI1: Modified triangular vegetation index
N: Nitrogen
NAOC: Normalized area over reflectance curve index
NDDA: Normalized difference of the double-peak areas
NDRE: Normalized difference red-edge index
NDVI: Normalized Difference Vegetation Index
NIR: Near infrared region
PSSRb: Pigment specific simple ratio chlorophyll b
*R*^2^: Coefficients of determination
RDVI: Re-normalized difference vegetation index
RE: Relative error
REFCA: Red edge reflectance curve area
RMSE: Root mean square error
RSDR: Right-side peak area
SAVI: Soil-adjusted vegetation index
SPAD: Soil and Plant Analyzer Development
SPP: Solar principal plane
TCI: Triangular chlorophyll index
TVI: Triangle vegetation index
UAV: Unmanned Aerial Vehicle
VI: Vegetation index
VIopt: Optimal vegetation index
VZA: View zenith angle.
